# Short-Term Ambient Air Ozone Exposure and Components of Metabolic Syndrome in a Cohort of Mexican Obese Adolescents

**DOI:** 10.3390/ijerph20054495

**Published:** 2023-03-03

**Authors:** Jorge Octavio Acosta Montes, Albino Barraza Villarreal, Blanca Gladiana Beltrán Piña, Karla Cervantes Martínez, Marlene Cortez Lugo, Isabelle Romieu, Leticia Hernández Cadena

**Affiliations:** 1 Facultad de Enfermería y Nutriología, Universidad Autónoma de Chihuahua, C. Escorza No. 900 Centro, Chihuahua 31000, Chihuahua, Mexico; 2 Instituto Nacional de Salud Pública, Av. Universidad No. 655, Col. Santa Maria Ahuacatitlán, Cuernavaca 62100, Morelos, Mexico

**Keywords:** air pollution, exposure assessment, environmental health policy, children’s health

## Abstract

Ambient air pollution is a major global public health concern; little evidence exists about the effects of short-term exposure to ozone on components of metabolic syndrome in young obese adolescents. The inhalation of air pollutants, such as ozone, can participate in the development of oxidative stress, systemic inflammation, insulin resistance, endothelium dysfunction, and epigenetic modification. Metabolic alterations in blood in components of metabolic syndrome (MS) and short-term ambient air ozone exposure were determined and evaluated longitudinally in a cohort of 372 adolescents aged between 9 to 19 years old. We used longitudinal mixed-effects models to evaluate the association between ozone exposure and the risk of components of metabolic syndrome and its parameters separately, adjusted using important variables. We observed statistically significant associations between exposure to ozone in tertiles in different lag days and the parameters associated with MS, especially for triglycerides (20.20 mg/dL, 95% CI: 9.5, 30.9), HDL cholesterol (−2.56 mg/dL (95% CI: −5.06, −0.05), and systolic blood pressure (1.10 mmHg, 95% CI: 0.08, 2.2). This study supports the hypothesis that short-term ambient air exposure to ozone may increase the risk of some components of MS such as triglycerides, cholesterol, and blood pressure in the obese adolescent population.

## 1. Introduction

Ambient air pollution is a major public health concern globally. Over 90% of the world’s population is estimated to live in zones where air pollutant concentrations exceed the World Health Organization guideline limits (WHO) [1]. In Mexico, ozone is found in concentrations higher than what is established as acceptable by the Official Mexican Standard, as estimated by the System of Atmospheric Monitoring of the Metropolitan Area of the Valley of Mexico (SIMAT) [2].

Several epidemiological studies have linked ambient air pollution with respiratory (chronic obstructive pulmonary disease, asthma, lung function decrease, and inflammatory airways) [3,4,5,6] and cardiovascular diseases [7,8,9] and lungs cancers [10,11]. These effects indicated that exposure to ambient air pollutants might cause events during the later stages of life and initiate chronic disease processes. However, the effects of air pollutants on the earlier stages of developing chronic diseases are less studied. Metabolic syndrome (MS) comprises a cluster of major modifiable risk factors for non-communicable diseases, including abdominal obesity, dyslipidemia, elevated blood pressure, and high glucose concentrations [12,13]. MS affects approximately 10–25% of the global population and its prevalence is rapidly increasing worldwide, and it has been suggested that the increase in the prevalence of MS is related with genetic factors, low physical activity, and an unhealthy lifestyle; however, the ambient air pollution could also be a risk factor for components of MS [14]. Under this context, the inhalation of air pollutants can participate in the development of oxidative stress, systemic inflammation [15,16], insulin resistance [17], endothelium dysfunction [18,19], and epigenetic modification. These negative responses can independently and/or interactively be involved with the development of cardiovascular symptoms, all of which are components in the diagnosis of MS. Previous epidemiological and experimental studies have explored the relationship between air pollution exposure and individual MS components [14,20,21]. However, the existing evidence focuses mainly on the adult population, one of the main reasons being the complexity of the diagnosis of metabolic syndrome in adolescents, so it becomes very important to study how exposures to environmental pollutants behave in metabolic disorders in this population group. Two previous epidemiological studies in humans investigated the relationship between air pollutants and MS, and both reported significant associations [14,22].

Additionally, a recent animal study showed that exposure to air pollutants (exposure to particulate matter) resulted in weight gain and cardiorespiratory and metabolic dysfunction [20]. More recently, studies in rats and humans reported that acute or short-term exposure to ozone, under controlled conditions, can lead to metabolic disturbances within hours or days since changes in the metabolome in blood samples were observed [21,23]. Even though these studies involving the metabolome have provided important information regarding new metabolites and the possible mechanisms of action of acute exposure to ozone, it is still necessary to evaluate the short-term effects on macromolecules derived from these metabolic processes more closely in population groups exposed to environmental fluctuations in ozone. Additionally, to our knowledge, no prior study has been conducted in Mexico to evaluate the association between short-term air pollution and MS in the adolescent population. Therefore, considering the current MS epidemic, the higher air pollution, and the scarcity of such an evaluation, our study would be the first to assess the relationships between short-term exposure to ozone and MS in Mexican adolescents.

## 2. Methods 

### 2.1. Design and Study Population

A dynamic cohort study of 415 adolescents living in the Metropolitan area of Mexico City was conducted from January 2006–August 2013. Participants were enrolled during the first three years and followed during one year on average (from 6 months until three years maximum) from when they attended the obesity clinic at Mexican Children’s Hospital Federico Gómez (HIM-FG), which provides health care to people from 0 to 18 coming from the entire metropolitan area of the Mexico City.

Mexico City is part of the metropolitan zone in the Valley of Mexico (MZVM), with nine million inhabitant; approximately 52% of the population are women, and 13.5% belong to the 10–19 age group [24]. This is considered the largest and most complex city in the country with high levels of traffic-related pollutants emissions [25].

The main objective of the cohort was to evaluate if weight loss improved lung function and reduced local inflammation in obese adolescents aged between 10 to 18 years old with and without asthma. All adolescents who met the criteria for being overweight and obese according to Cole et al. [26] and agreed to participate in the study were included in a program of nutritional, physical, and psychological orientation to improve their “healthy” life. Adolescents were given recommendations to increase their physical activity for half an hour per day, and a nutritionist give orientation for a healthier diet based on the WHO recommendations according to age and sex (60% of carbohydrate, 20% proteins, and 20% fat).

All participating adolescents signed an informed consent letter in addition to the consent letter from both parents. The protocol was approved by the ethics committees of the Children’s Hospital of Mexico Federico Gómez and the National Institute of Public Health. The adolescents were cited for the first time for an evaluation, where they had a clinical history and blood samples were taken to evaluate the metabolic profile (cholesterol, triglycerides, high- and low-density lipoproteins, uric acid, glucose). Participants were cited every three months for taking blood samples and for receiving dietary guidelines and the questionnaires on the frequency of food consumption and physical activity were applied. For every 15 days during the first three months and every month during the following months up until one year, the child received psychological attention through trained personnel.

As part of this cohort, and preserving the longitudinal character of the base study, we selected a subsample of 372 adolescents aged 9 to 19 years, diagnosed with overweight and obesity, in which metabolic alterations in blood were evaluated every three months.

### 2.2. Data Collection

#### 2.2.1. Components of Metabolic Syndrome Evaluation and Other Measures

To determine the biochemical parameters of metabolic syndrome, blood samples were taken by trained personnel, and a sample of approximately 7 mL was extracted from a vein of the participant’s arm and duly safeguarded to maintain its integrity. The sample was obtained during the first hours of the morning, asking the adolescent to come fasting and in optimal hydration conditions. The sample was centrifuged and separated into 2.5 mL vials, and then it was sent to freeze for storage and subsequent analysis. All the extractions were conducted according to the manufacturer’s instructions. 

Blood pressure was taken through a baumanometer by trained personnel. The adolescent was left with 15 min of rest, and the shot was taken twice to obtain an average of the measurements and have a more accurate value. Information on anthropometric measures was obtained from participants at the baseline and during the follow-up period. Each participant was weighted while wearing light clothing and standing without shoes on a calibrated platform scale (brand healthometer, model 402 KL, with a minimum capacity of 100 g). The height (cm) was obtained using a Holtain Limited Crymych, Dyfec stadiometer barefoot on a flat surface, making a right angle with the vertical bar of the stadiometer and asking each patient to inhale before sliding the headboard over the top point of their head. The BMI (BMI = weight/height^2^) was calculated to indirectly quantify body fat, considering the following cut-off points: 20, 25, and 30, corresponding to the categories of normal weight, overweight, and obesity, according to Cole. 

The cut-off points for the parameters related (triglycerides, HDL cholesterol, and fasting glucose) to the diagnosis of MS were those established by the FID [27]. A participant was considered as positive for MS if he had a waist circumference greater than the 90th percentile or the threshold or, failing that, the condition of overweight or obesity according to the body mass index, plus two criteria of the following: (1) triglycerides levels > 150 mg/dL, (2) HDL cholesterol levels < 40 mg/dL, (3) systolic blood pressure > 130 mmHg, (4) diastolic blood pressure > 85 mmHg, and (5) fasting glucose levels >to 100 mg/dL. 

The parameters of the MS were managed in two forms; in the first, the values for each component were considered continuously and each one was handled as an individual variable. In the second, a joint variable (yes and no) was explored and constructed from the different parameters that make up the MS based on the definition of the FID [27]. 

#### 2.2.2. Exposure Assessment

All the information related to the air pollutants, as well as the information of meteorological variables (direction and wind speed, humidity, and temperature), were obtained through the Atmospheric Monitoring System of the Metropolitan Zone of the Valley of Mexico (SIMAT), which makes measures continuously for 365 days of the year. Currently, in the MZVM, atmospheric monitoring networking (AMN) has 40 air quality monitors.

The daily exposures to ozone and the other pollutants were constructed considering two important times of the day, the shift attended at school (morning: 7:00 to 14:00 h and evening: 15:00 to 19 h), and the remaining hours were assigned to the corresponding exposure to the home address. According to the above, hourly averages were used to obtain a maximum of 24 h hours or a daily maximum (1-h maximum): a maximum of 8 h according to school or home address was estimated per adolescent once their exposure diary was constructed, and delays of 1 up to 15 days prior to the visit to the blood sample collection were recorded.

The exposure to O_3_ was assigned using a geographic information system (GIS), which considered the distance between the monitor and the area where both the home and school of the participants were located, based on their address and zip code, estimating the closest monitor’s exposure either to the school or home according to the school shift. Additionally, during the study period (specifically in 2010), the AMN made some changes both in the location of some monitors and in the placement or elimination of others; therefore, these were considered in the assigning of the closet monitor.

### 2.3. Other Variables

#### 2.3.1. Diet

The dietary intake was assessed using a validated food frequency consumption questionnaire, which indicates how many times a week and how many servings of food the participant consumed per day. This report was intended to represent dietary consumption during the three months that elapse between one measurement and another. The questionnaire was applied by trained nutritionists and was answered by the adolescent, supported by the person who accompanied them to the consultation. To obtain the consumption of kilocalories consumed in one day, the portions of each of the foods that the participant consumed during a week were calculated to then obtain a daily average of carbohydrates, lipids, and proteins; the calculation of micronutrients consumed during a day (antioxidants: vitamin C and vitamin E) was conducted in the same way. The nutritional contribution of each of the foods was calculated based on the reference values of food composition established by the Tables for Practical Use of Foods of Greater Consumption in its third edition [28], as well as by what is established in the Mexican System of Equivalent Foods in its third edition.

#### 2.3.2. Physical Activity

Physical activity was categorized as mild, moderate, and intense through a short physical activity questionnaire assessed according to each adolescent visit. The questionnaire consisted of 6 questions evaluating practice about physical activity in the last 7 days and the time it took: vigorous (running, swimming, riding a bike, or playing in some team), moderate (quick walk or jog of 20 min or more), or light (walking 20 min). Additionally, it explores the time spent remaining sitting in front of a television, a computer, or playing video games. The questionnaire was applied by trained personnel and was answered by the adolescent, supported by the person who accompanied them to the consultation.

### 2.4. Statistical Analysis

An exploratory analysis of the information was carried out, where each variable’s quality and distribution were evaluated. Added to this, the minimum and maximum ranges of the variables were analyzed to know if there was any extreme value that would affect the distribution, eliminating two low main criteria and entry to those that were not biologically plausible, and ensuring that these values do not represent more than 5% of the total of the values within the variable. The short-term association of ozone exposure with the metabolites outcomes was evaluated using linear mixed-effects models, considering the ID of the participant as a random intercept, and using models for continuous and binary responses (only for the metabolic syndrome condition). We also evaluated as potential confounders the physical activity, BMI, antioxidant intake (vitamin C and vitamin E), asthma presence, kilocalories consumed, and meteorological variables, considering only those that were significant in the final model. Likewise, statistical significance was evaluated from the inclusion or exclusion of each variable to elucidate how the coefficient was affected by time, with the interest of finding the best estimate of the model with the least number of variables. 

A mixed-effects model with random intercept was used since some variables had different measurements over time; however, there were some more that were maintained through the study. Within the mixed models, the short-term exposure was evaluated based on tertiles, leaving the lowest tertile as a reference category for the remaining two. The cut-off points for each tertile were different depending on the number of days before the sample was taken, within which the exposure (lags) was considered. Lag days from 0 to 15 were considered, according to that reported in the previous literature, in which multiple lags due to short-term ozone exposure are evaluated for cardio metabolic risk [22]. To prevent the results from being biased via exposure to other pollutants, especially particulate matter, which has been widely evaluated as associated with ischemic disease and cardiovascular risk, we adjusted the model by adjusting for PM_2.5_ concentrations of the same lag as for ozone using the maximum of 24 h. A stratified analysis was performed with the conditions of asthmatic or non-asthmatic; however, no statistically significant association was obtained. All the statistical analysis was carried out using the statistical package STATA 14.0.

## 3. Results

The mean age of participants was 12.8 years (SD = 2.1 years). More than half of the subjects were males (56.5%). The characteristics of the study participants are summarized in Table 1. Based on the main definition of MS for the study, the prevalence of MS was 10.0%. In Table 2, we can see the results related to the parameters are indicative of MS. We found that 45% of the participants were obese; in terms of fasting glucose levels, only 8% of the participants presented values above what was considered normal, 40% had HDL cholesterol levels below established limits, 35% had triglyceride levels above the cut-off point of normality, only 3% of the participants presented high blood pressure of both the systolic and the diastolic kind, and 9% of participants met criteria for the classification of metabolic syndrome in the baseline data.

Table 3 summarizes the descriptive statistics of air pollution concentrations and meteorological variables based on the geographical area of the Metropolitan Area of the Valley of Mexico. We found that the ozone concentration is lower in the northern part and must increase concentration in the areas further south. This coincides with a slight increase in the average temperature for the central and southern areas, which, added to the wind and relative humidity conditions, means that the population living in these areas is exposed to slightly higher concentrations than the rest.

The association between ozone ambient air concentrations and components of MS are summarized in Table 4. In general, we observed a statistically significant tertile trend increase between tertile exposure to ozone and some of the parameters related to MS. The lags that showed a statistically significant association were those corresponding to 2, 7, 8, 11, and 13 days prior to the visit. We found an increase in triglyceride levels of 20.24 mg/dL (95% CI: 9.54, 30.95) for ozone exposure on lag day 2, as well as an increase of 12.55 mg/dL (95% CI: 1.44, 23.66) on lag day 11 for those in the third tertile relative to the lowest tertile for ozone exposure. Comparing in a similar way for those in the third tertile with the lowest tertile, regarding HDL cholesterol, a decrease in blood concentration of −2.56 mg/dL (95% CI: −5.06, −0.05) was observed in the ozone exposure on lag day 7; however, on lag day 8 of ozone exposure, the decrease was more significant: −3.46 mg/dL (95% CI: −5.96, −0.95). Blood pressure in general showed a statistically significant change on lag day 13, increasing by 1.14 mmHg (95% CI: 0.08, 2.2) in systolic blood pressure, while for diastolic blood pressure, the increase was 0.91 mmHg (95% CI: −0.03, 1.86), this being marginally significant. According to the adolescents who had the characteristics of metabolic syndrome, we observed an OR of risk of 2.23 (95%CI: 1.10, 4.56) and 1.99 (95%CI: 0.93, 4.25) for third tertile ozone exposure on lag days 2 and 3, respectively. We also observed increased risk with lag 15. 

All models were carried out using the mixed-effects model with random intercept adjusted by physical activity, BMI, antioxidant intake (Vitamin C and Vitamin E), and asthma presence. Meteorological variables were tested as adjustment variables, but these were not statistically significant, nor did either change the sense of association between ozone and the metabolite evaluated (Table 4). Additionally, we evaluated these models by adjusting PM_2_._5_ concentrations (as a continuous variable, not in tertiles) using the same lag time than ozone. The results are shown in Table 4. In most cases, the significance of ozone remains. PM_2_._5_ was significant principally at lag 4 (Supplementary Materials).

## 4. Discussion

In this cohort study, we found that short-term ambient air exposure to ozone was significantly associated with an increase in some parameters related to MS in young populations. Although exposure to outdoor ambient levels of PM_2_._5_, NO_2,_, and O_3_ has been associated with asthma, respiratory diseases, and respiratory symptoms in children mainly, there are few previous longitudinal studies that have studied this type of association in populations between this age range and particularly in obese adolescents; to our knowledge, this is the first study to evaluate these effects in a low–middle income country.

We found associations in different exposure lags between 2 and 14 days in most of the components of MS, which could lead adolescents, given their condition of obesity, to a greater risk of being classified as positive for metabolic syndrome based on the classification of the FID. The fact that some components were related to 2-day lags and more lag days may also be due to the possible correlation that exists in the pollutants due to weekly cyclical trends. In one study in rats, it was reported that short-term exposure to ozone can lead to higher levels of leptin and blood glucose, as well as other changes in metabolites involved with the metabolism of glucose, lipids, and amino acids after subjecting a group of rats for a few hours to high ozone concentrations [21]. Subsequently, the same researchers showed, in a controlled human study, that after short-term exposure to ozone circulating lipid metabolites were altered as a result of changes in metabolism, giving rise to the saturation of certain metabolic pathways [23], suggesting alterations in membrane phospholipids linked to proinflammatory mechanisms due to ozone. We believe that if these effects are sustained for longer periods of time, they can trigger permanent damage to the metabolic system or even the immune system. One study that indicated that long-term exposure to ambient air pollutants may increase the risk of metabolic syndrome, especially among males, results that are consistent with our findings; however, their results come from a cross-sectional study conducted in the adult population [29]. Another study reported a positive association between ozone exposures and type 1 diabetes as well as alterations in plasma lipid profiles and lower levels of glucagon-like peptide one after exposure to highly polluted air, respectively, indicating that sub-chronic exposure to ozone-induced beta-cell dysfunction may secondarily contribute to other tissue-specific metabolic alterations, due to an impaired regulation of glucose, lipid, and protein metabolism in young adult rats [30]. Similarly, an increase in oxidative stress has been observed in rats exposed to high concentrations of ozone, leading to mitochondrial DNA damage as well as an endothelial vascular decrease in nitric oxide synthetase, producing a significant increase in atherogenesis in comparison with rats that are exposed to filtered air, results that provide further experimental evidence for the possible link between air pollution and MS [31].

There are different hypotheses that describe the possible biological mechanisms that support our findings. Although these mechanisms are still not completely clear, it has been described that air pollutants may perturb autonomic nervous system balance by activating afferent pulmonary autonomic reflexes; additionally, when ozone enters the body through the respiratory tract, it reacts with the existing biomolecules in the fluid that covers the lungs, generating highly reactive products that enter the bloodstream, promoting cascade inflammation mechanisms that can lead to damage in the cardiac vasculature, which in turn can induce arrhythmias, myocyte reduction, contractility, and decreased coronary blood flow due to acute vasoconstriction, which can increase blood pressure [15,22,32]. Similarly, it has been described that exposure to air pollutants may induce the generation and release of endogenous pro-inflammatory mediators and vasculo-active molecules, which can disrupt insulin signaling and impair vasorelaxation [30,33]. The oxidative stress promotes the activation of Nrf2, the heat shock protein 70, NF-kB, increases the expression of a variety of proinflammatory cytokines (TNF-alpha and interleukin 1β), chemokines (Interleukin 8), and adhesion genes, and, finally, some studies report that air pollution exposure is associated with abnormal methylation levels of global DNA and specific genes involved in glucose homeostasis and lipid metabolism pathways [34,35]. One study in 2015 proposes that short-term exposure to ozone can increase circulating cortisol and is reflective of an activation of a neurohormonal-mediated stress response, likely through the activation of the HPA axis and altered lipid metabolic processes stimulating the adipose lipolysis of triglyceride stores and being liberated into the circulation. The increased lysolipids, likely released from the hydrolysis of cellular and membrane phospholipids, and serum polyunsaturated fatty acids in ozone-exposed humans may be linked to proinflammatory mechanisms due to ozone exposure and showed elevated circulating metabolites of β-oxidation and ω-oxidation; overall, this study demonstrates that ozone exposure in humans is associated with increased release of stress hormones causing lipolysis, as in rodents [21].

Some limitations must be considered when interpreting our results. First, daily variations in ozone air pollutant exposure were evaluated through the daily records of the fixed central monitoring locations (RAMA). The temporal variations in each adolescent’s exposure were assumed to follow those at the central monitoring site, and we did not obtain detailed information about the time spent by each participant; instead, we presumed that exposure was primarily associated with the amount of time spent in the outdoors. To strengthen the validity of this assumption, each adolescent was assigned to the monitoring site closest to his or her home or school by means of a spatial GIS, providing greater variability in the data. Second, the possibility that the results were a consequence of the poor control of confounders, such as socioeconomic status, however, is unlikely because all our participants came from the same study area and attended the same public school system; also, the design of the study excluded women with a high risk of pregnancy and/or pre-existing illness and the models were adjusted for potential confounders.

On the other hand, within the main strengths of this study, we can mention that it is a longitudinal study with a good participation rate (89% of participants from the cohort entered this evaluation). Additionally, the fact of having valuable information on other variables of importance at different times of the follow-up gives greater support to the findings. In this sense, being a cohort study, we can highlight that we had the possibility of observing that the exposure precedes the event, and our results were based on an observational analysis of the cohort; also, we used mixed linear multivariate models to account for the strong patterns of association among the outcome and exposure variables and for the control of confounders.

In this study, we adjusted for variation in physical activity, an important prediction factor in metabolic syndrome. In previous studies [23], it has been evaluated that certain metabolic changes due to ozone exposure can vary after exercising. Even so, we did not find significant differences when it was explored in a stratified way between adolescents who did vigorous activity and those who did not. Perhaps in a future study, it would be advisable to expand the sample size and evaluate these activities more precisely, as well as to support the results presented in this study.

## 5. Conclusions

Our data show that short-term ambient air ozone exposure is associated with the components of MS. These adverse effects were observed in a longitudinal setting in a free-living population, more specifically in a cohort of obese adolescents. These results could have significant public health policy implications, and derived that metabolic syndrome was defined by a combination of various cardiovascular disorders (hypertension, dyslipidemia), elevated triglycerides and lowered high-density lipoprotein cholesterol, raised fasting glucose, obesity, and is associated with systemic inflammation and increases the risk of cardiovascular disease in the early stages of life.

## Figures and Tables

**Table 1:** Main characteristics of the study participants (adolescents obese, México City, *n* = 372).

Variable	Description
Sex, *n* (%)	
Female	162 (43.5)
Male	210 (56.5)
Age (years)	
Mean (SD)	12.8 (2.1)
Range	9.3 a 19.6
Schooling, *n* (%)	
Elementary	189 (51.4)
Secondary	149 (40.2)
High school	34 (8.4)
Mother’s age at birth of the child (years)	
Mean (SD)	26.2 (6.1)
Range	15 a 49
Birth weight (kg)	
Mean (SD)	2.84 (0.71)
Range	1.2–5.5
School shift, *n* (%)	
Morning	289 (79.6)
Evening	83 (20.4)
Asthma diagnosis, *n* (%)	
Non	214 (57.5)
Yes	158 (42.5)
Number of visits, *n* (%) *	
One visit	104 (28.5)
From 2 to 9	255 (66)
From 10 to 16	13 (5.5)

Variables were evaluated at the baseline visit. * Additional visits to the basal.

**Table 2:** Description of baseline MS components in obese adolescents, México City (*n* = 372).

Component	*n*	Risk Factor	Mean (SD **)	Min–Max
Body mass index (weight/hight^2^)	151	Yes	33.9 (4.0)	30–52.7
	221	Non	26.0 (1.9)	18.7–29.8
* Fasting glucose (mg/dL)	28	Yes	120.3 (28.3)	100–208
	344	Non	86.4 (6.7)	69–99
* HDL Cholesterol (mg/dL)	134	Yes	33.3 (5.7)	10–40
	238	Non	52.8 (17.7)	41–173
* Triglycerides (mg/dL)	138	Yes	227.7 (87.1)	150–577
	234	Non	95.8 (29.8)	30–149
* Systolic Blood pressure (mmHg)	11	Yes	132.5 (4.5)	130–140
	361	Non	105.8 (6.4)	80–122
* Diastolic Blood pressure(mmHg)	11	Yes	89.8 (4.5)	85–100
	360	Non	67.0 (6.1)	50–80
* Metabolic syndrome (MS)	33	Yes	8.9%	-
	339	Non	91.1%	-

* Cut-off points established according to IDF: fasting glucose higher than 100 mg/dL, HDL cholesterol less than 40 mg/dL, triglycerides more than 150 mg/dL concentration, systolic and diastolic blood pressure higher or equal than 130 and 85 mmHg, respectively, an MS in the presence of obesity and at least two risk factors. ** Standard deviation.

**Table 3:** Meteorological variables and pollutants distribution from 2010 to 2013, Mexico City.

Variable	Geographical Area				
	Northeast	Northwest	Center	Southeast	Southwest
	Mean (SD)	Mean (SD)	Mean (SD)	Mean (SD)	Mean (SD)
Ozone (ppm)	0.07 (0.03)	0.08 (0.03)	0.09 (0.03)	0.08 (0.03)	0.08 (0.03)
PM_10_ (μg/m^3^)	80.70 (85.71)	86.51 (62.07)	76.15 (43.98)	104.51 (65.74)	80.05 (42.39)
PM_2_._5_ (μg/m^3^)	47.13 (26.01)	55.06 (46.16)	52.28 (18.6)	63.49 (43.91)	44.26 (20.34)
NO_2_ (ppb)	0.05 (0.02)	0.05 (0.02)	0.07 (0.03)	0.06 (0.02)	0.06 (0.02)
Temperature (°C)	23.94 (3.81)	23.88 (3.20)	25.07 (3.13)	25.1 (3.99)	23.1 (3.48)
Humidity (%)	72.51 (16.16)	76.29 (14.65)	78.39 (14.20)	74.52 (15.26)	75.25 (15.96)
Wind speed (m/s)	4.18 (1.09)	3.98 (0.96)	3.29 (0.91)	3.26 (1.44)	3.1 (0.97)

Note: Data show 1-h maximum in 24 h.

**Table 4:** Association between metabolic syndrome and components of MS with short-term exposure to ozone at different lags of time in obese adolescents, México City.

	Model Non-Adjusted by PM2.5		* Model Adjusted by PM2.5	
	Coefficient (CI 95%)	*p* Value	Coefficient (CI 95%)	*p* Value
Triglycerides mg/dL				
Lag 2				
Tertile 2 (0.065–0.092)	9.11 (−1.50, 19.73)	0.092	11.38 (−0.25, 23.02)	0.055
Tertile 3 (0.093–0.179)	20.24 (9.54, 30.95)	0.001	21.51 (9.35, 33.66)	0.001
Triglycerides mg/dL				
Lag 11				
Tertile 2 (0.066–0.094)	4.64 (−6.19, 15.47)	0.401	3.62 (−8.4, 15.64)	0.555
Tertile 3 (0.092–0.177)	12.55 (1.44, 23.66)	0.027	14.30 (1.89, 26.7)	0.024
HDL cholesterol mg/dL				
Lag 7				
Tertile 2 (0.068–0.091)	−0.22 (−2.69, 2.25)	0.861	0.93 (−8.41, 15.64)	0.511
Tertile 3 (0.092–0.177)	−2.56 (−5.06, −0.05)	0.045	−0.58 (−3.45, 2.92)	0.692
HDL cholesterol mg/dL				
Lag 8				
Tertile 2 (0.067–0.091)	−3.94 (−6.40, −1.49)	0.002	−4.02 (−6.72, −1.31)	0.004
Tertile 3 (0.092–0.178)	−3.46 (−5.96, −0.95)	0.007	−2.82 (−5.58, −0.07)	0.044
B.P. systolic mmHg				
Lag 13				
Tertile 2 (0.071–0.096)	0.88 (−0.15, 1.92)	0.097	0.93 (−1.85, 3.72)	0.082
Tertile 3 (0.097–0.188)	1.14 (0.08, 2.21)	0.035	−0.58 (−3.45, 2.29)	0.082
B.P. diastolic mmHg				
Lag 14				
Tertile 2 (0.071–0.096)	0.83 (−0.92, 1.77)	0.077	1.02 (−0.12,2.17)	0.092
Tertile 3 (0.097–0.188)	0.91 (−0.03, 1.86)	0.058	1.05 (−0.13, 2.24)	0.039
MS (Yes/Non)				
Lag 2				
Tertile 2 (0.065–0.092)	1.12 (0.52, 2.41)	0.767	0.42 (0.44, 2.28)	0.999
Tertile 3 (0.093–0.179)	2.23 (1.10, 4.56)	0.027	1.00 (0.91, 4.29)	0.087
MS (Yes/Non)				
Lag 3				
Tertile 2 (0.068–0.094)	2.43 (1.15, 5.14)	0.020	1.97 (0.89, 4.37)	0.096
Tertile 3 (0.095–0.211)	1.99 (0.93, 4.25)	0.076	1.66 (0.73, 3.73)	0.225
MS (Yes/Non)				
Lag 15				
Tertile 2 (0.066–0.094)	1.85 (0.87, 3.94)	0.109	2.33 (1.02, 5.35)	0.046
Tertile 3 (0.095–0.184)	2.66 (1.24, 5.69)	0.012	2.91 (1.27, 6.66)	0.011

Note: Linear models (mixed-effects model with random intercept) adjusted by physical activity, BMI, antioxidant intake (Vitamin C and Vitamin E), and asthma presence; *n* = 372 averages of evaluations per participant = 3.4, 2 visits minimum and 16 visits maximum with differences between each visit of 3 months minimum). Ozone unit measurement was made using parts per million (ppm) and categorized in tertiles of exposure. Blood pressure (BP). * Models were adjusted using PM_2_._5_ as the continuum variable.

## Data Availability

Not applicable.

## Supplementary Materials

The following supporting information can be downloaded at: https://www.mdpi.com/article/10.3390/ijerph20054495/s1, Figure S1. Association between metabolic syndrome (Yes/No) and short term exposure to ozono at differents lags of time (1 to 15 days) in adolescents obese, Mexico City; Figure S2. Association between tryglicerides (mg/dl) and short term exposure to ozono at differents lags of time (1 to 15 days) in adolescents obese, Mexico City; Figure S3. Association between HDL cholesterol (mg/dl) and short term exposure to ozono at differents lags of time (1 to 15 days) in adolescents obese, Mexico City; Figure S4. Association between systolic blood pressure (mmHg) and short term exposure to ozono at differents lags of time (1 to 15 days) in adolescents obese, Mexico City; Figure S5. Association between diastolic blood pressure (mm Hg) and short term exposure to ozono at differents lags of time (1 to 15 days) in adolescents obese, Mexico City.

## References

[1] World Health Organization Nueve de Cada Diez Personas de Todo El Mundo Respiran Aire Contaminado Sin Embargo, Cada Vez Hay Más Países Que Toman Medidas. https://www.who.int/es/news/item/02-05-2018-9-out-of-10-people-worldwide-breathe-polluted-air-but-more-countries-are-taking-action.

[2] Instituto Nacional de Ecología y Cambio Climático (INECC) (2018). Informe Nacional de Calidad Del Aire 2017, México.

[3] Doiron D., de Hoogh K., Probst-Hensch N., Fortier I., Cai Y., de Matteis S., Hansell A.L. (2019). Air Pollution, Lung Function and COPD: Results from the Population-Based UK Biobank Study. Eur. Respir. J..

[4] Romieu I., Barraza-Villarreal A., Escamilla-Nuñez C., Almstrand A.C., Diaz-Sanchez D., Sly P., Olin A.C. (2008). Exhaled Breath Malondialdehyde as a Marker of Effect of Exposure to Air Pollution in Children with Asthma. J. Allergy Clin. Immunol..

[5] Barraza-Villarreal A., Escamilla-Nuñez M.C., Hernández-Cadena L., Texcalac-Sangrador J.L., Sienra-Monge J.J., Del Río-Navarro B.E., Cortez-Lugo M., Sly P.D., Romieu I. (2011). Elemental Carbon Exposure and Lung Function in Schoolchildren from Mexico City. Eur. Respir. J..

[6] Vogel C.F.A., Van Winkle L.S., Esser C., Haarmann-Stemmann T. (2020). The Aryl Hydrocarbon Receptor as a Target of Environmental Stressors—Implications for Pollution Mediated Stress and Inflammatory Responses. Redox Biol..

[7] Huang K., Liang F., Yang X., Liu F., Li J., Xiao Q., Chen J., Liu X., Cao J., Shen C. (2019). Long Term Exposure to Ambient Fine Particulate Matter and Incidence of Stroke: Prospective Cohort Study from the China-PAR Project. BMJ.

[8] Yang B.Y., Qian Z., Howard S.W., Vaughn M.G., Fan S.J., Liu K.K., Dong G.H. (2018). Global Association between Ambient Air Pollution and Blood Pressure: A Systematic Review and Meta-Analysis. Environ. Pollut..

[9] Chen H., Burnett R.T., Kwong J.C., Villeneuve P.J., Goldberg M.S., Brook R.D., van Donkelaar A., Jerrett M., Martin R.V., Kopp A. (2014). Spatial Association between Ambient Fine Particulate Matter and Incident Hypertension. Circulation.

[10] Hamra G.B., Laden F., Cohen A.J., Raaschou-Nielsen O., Brauer M., Loomis D. (2015). Lung Cancer and Exposure to Nitrogen Dioxide and Traffic: A Systematic Review and Meta-Analysis. Environ. Health Perspect..

[11] Xue Y., Wang L., Zhang Y., Zhao Y., Liu Y. (2022). Air Pollution: A Culprit of Lung Cancer. J. Hazard. Mater..

[12] Gutiérrez J.P., Rivera-Dommarco J., Shamah-Levy T., Villalpando-Hernández S., Franco A., Cuevas-Nasu L., Romero-Martínez M., Hernández-Ávila M. (2012). Encuesta Nacional de Salud y Nutrición 2012. Resultados Nacionales.

[13] Bovolini A., Garcia J., Andrade M.A., Duarte J.A. (2021). Metabolic Syndrome Pathophysiology and Predisposing Factors. Int. J. Sports Med..

[14] Wallwork R.S., Colicino E., Zhong J., Kloog I., Coull B.A., Vokonas P., Schwartz J.D., Baccarelli A.A. (2017). Ambient Fine Particulate Matter, Outdoor Temperature, and Risk of Metabolic Syndrome. Am. J. Epidemiol..

[15] Romieu I., Castro-Giner F., Kunzli N., Sunyer J. (2008). Air Pollution, Oxidative Stress and Dietary Supplementation: A Review. Eur. Respir. J..

[16] Miller M.R. (2014). The Role of Oxidative Stress in the Cardiovascular Actions of Particulate Air Pollution. Biochem. Soc. Trans..

[17] Rajagopalan S., Brook R.D. (2012). Air Pollution and Type 2 Diabetes: Mechanistic Insights. Diabetes.

[18] Lederer A.M., Fredriksen P.M., Nkeh-Chungag B.N., Everson F., Strijdom H., de Boever P., Goswami N. (2021). Cardiovascular Effects of Air Pollution: Current Evidence from Animal and Human Studies. Am. J. Physiol. Hear. Circ. Physiol..

[19] Miller M.R., Newby D.E. (2020). Air Pollution and Cardiovascular Disease: Car Sick. Cardiovasc. Res..

[20] Wei Y., Zhang J., Li Z., Gow A., Chung K.F., Hu M., Sun Z., Zeng L., Zhu T., Jia G. (2016). Chronic Exposure to Air Pollution Particles Increases the Risk of Obesity and Metabolic Syndrome: Findings from a Natural Experiment in Beijing. FASEB J..

[21] Miller D.B., Karoly E.D., Jones J.C., Ward W.O., Vallanat B.D., Andrews D.L., Schladweiler M.C., Snow S.J., Bass V.L., Richards J.E. (2015). Inhaled Ozone (O3)-Induces Changes in Serum Metabolomic and Liver Transcriptomic Profiles in Rats. Toxicol. Appl. Pharmacol..

[22] Goodman J.E., Prueitt R.L., Sax S.N., Pizzurro D.M., Lynch H.N., Zu K., Venditti F.J. (2015). Ozone Exposure and Systemic Biomarkers: Evaluation of Evidence for Adverse Cardiovascular Health Impacts. Crit. Rev. Toxicol..

[23] Miller D.B., Ghio A.J., Karoly E.D., Bell L.N., Snow S.J., Madden M.C., Soukup J., Cascio W.E., Gilmour M.I., Kodavanti U.P. (2015). Ozone Exposure Increases Circulating Stress Hormones and Lipid Metabolites in Humans. Am. J. Respir. Crit. Care Med..

[24] Instituto Nacional de Estadística y Geografía Número de Habitantes. Ciudad de México.

[25] Dirección General de Calidad Del Aire, Dirección de Proyectos de Calidad Del Aire (2021). Secretaría del Medio Ambiente de la Ciudad de México Inventario de Emisiones de La Zona Metropolitana Del Valle de México 2018.

[26] Cole T.J., Bellizzi M.C., Flegal K.M., Dietz W.H. (2000). Establishing a Standard Definition for Child Overweight and Obesity Worldwide: International Survey. Br. Med. J..

[27] Zimmet P., Alberti G., Kaufman F., Tajima N., Silink M., Arslanian S., Wong G., Bennett P., Shaw J., Caprio S. (2007). El Síndrome Metabólico En Niños y Adolescentes: El Consenso de La FID. Diabetes Voice.

[28] Chávez A., Ledesma J., Mendoza E., Calvo C., Castro M., Ávila A., Sánchez C., Pérez-Gil F. (2014). Tablas de Uso Práctico Del Valor Nutritivo de Los Alimentos de Mayor Consumo En México.

[29] Yang B.Y., Qian Z.M., Li S., Fan S., Chen G., Syberg K.M., Xian H., Wang S.Q., Ma H., Chen D.H. (2018). Long-Term Exposure to Ambient Air Pollution (Including PM1) and Metabolic Syndrome: The 33 Communities Chinese Health Study (33CCHS). Environ. Res..

[30] Miller D.B., Snow S.J., Henriquez A., Schladweiler M.C., Ledbetter A.D., Richards J.E., Andrews D.L., Kodavanti U.P. (2016). Systemic Metabolic Derangement, Pulmonary Effects, and Insulin Insufficiency Following Subchronic Ozone Exposure in Rats. Toxicol. Appl. Pharmacol..

[31] Chuang G.C., Yang Z., Westbrook D.G., Pompilius M., Ballinger C.A., White C.R., Krzywanski D.M., Postlethwait E.M., Ballinger S.W. (2009). Pulmonary Ozone Exposure Induces Vascular Dysfunction, Mitochondrial Damage, and Atherogenesis. Am. J. Physiol. Lung Cell. Mol. Physiol..

[32] Dabass A., Talbott E.O., Rager J.R., Marsh G.M., Venkat A., Holguin F., Sharma R.K. (2018). Systemic Inflammatory Markers Associated with Cardiovascular Disease and Acute and Chronic Exposure to Fine Particulate Matter Air Pollution (PM2.5) among US NHANES Adults with Metabolic Syndrome. Environ. Res..

[33] Ayer A., Fazakerley D.J., James D.E., Stocker R. (2022). The Role of Mitochondrial Reactive Oxygen Species in Insulin Resistance. Free Radic. Biol. Med..

[34] Peng C., Bind M.C., Colicino E., Kloog I., Byun H.M., Cantone L., Trevisi L., Zhong J., Brennan K., Dereix A.E. (2016). Particulate Air Pollution and Fasting Blood Glucose in Nondiabetic Individuals: Associations and Epigenetic Mediation in the Normative Aging Study, 2000–2011. Environ. Health Perspect..

[35] Wang C., Chen R., Cai J., Shi J., Yang C., Tse L.A., Li H., Lin Z., Meng X., Liu C. (2016). Personal Exposure to Fine Particulate Matter and Blood Pressure: A Role of Angiotensin Converting Enzyme and Its DNA Methylation. Environ. Int..

