# Supplementary material for: Short-Term Ambient Air Ozone Exposure and Components of Metabolic Syndrome in a Cohort of Mexican Obese Adolescents
Source: Int J Environ Res Public Health. 2023 Mar 3;20(5):4495. doi: 10.3390/ijerph20054495 (PMC10001840; doi:10.3390/ijerph20054495)
Supplement: Supplementary file 1 [file ijerph-20-04495-s001.zip › ijerph-2197873-supplementary.pdf]

Supplementary material

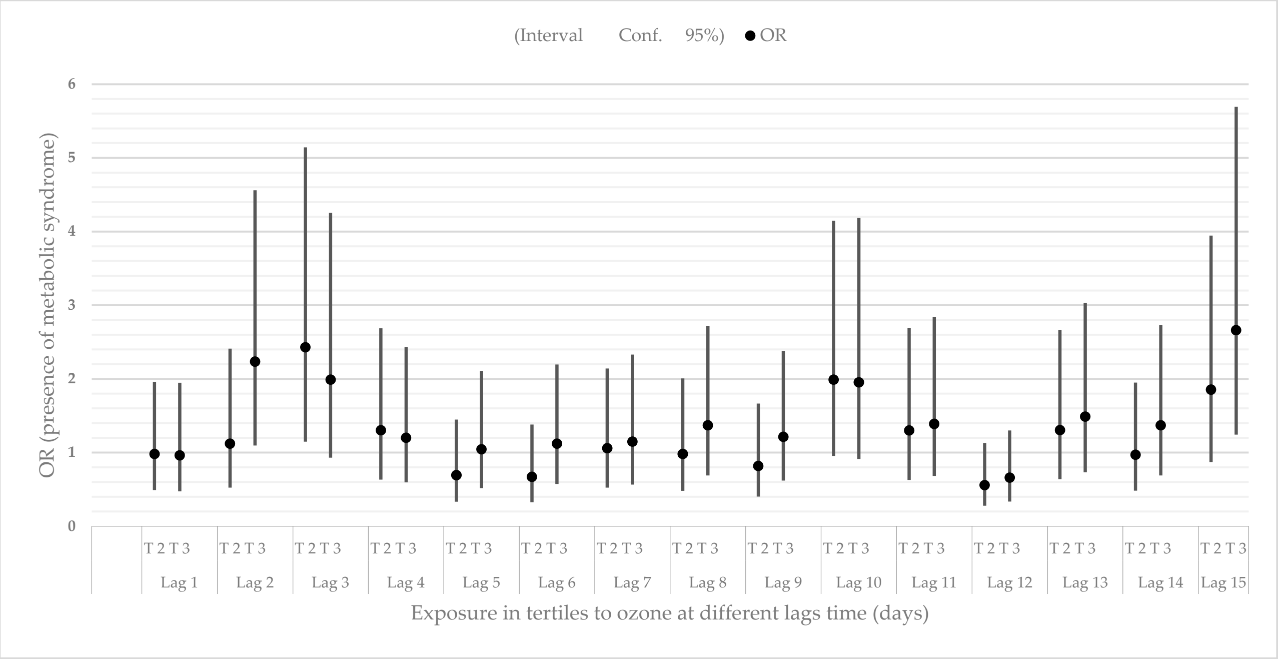

Figure S1. Association between metabolic syndrome (Yes/No) and short term exposure to ozono at differents lags of time (1 to 15 days) in adolescents obese, México City.

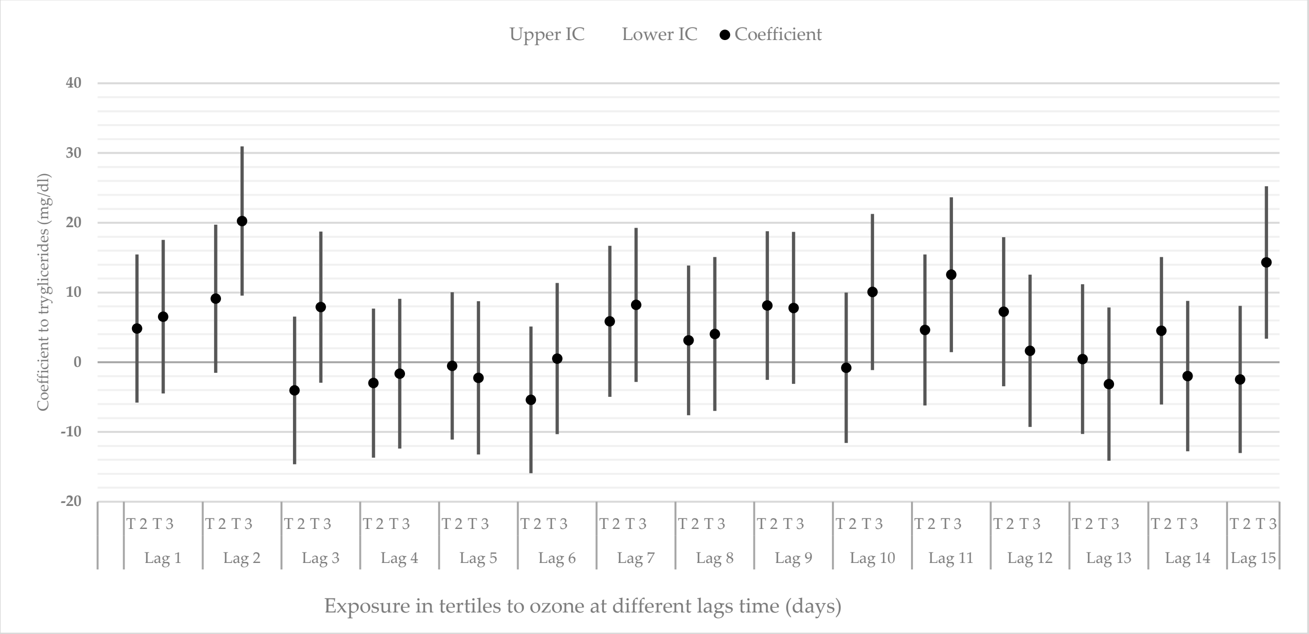

Figure S2. Association between tryglicerides (mg/dl) and short term exposure to ozono at differents lags of time (1 to 15 days) in adolescents obese, México City.

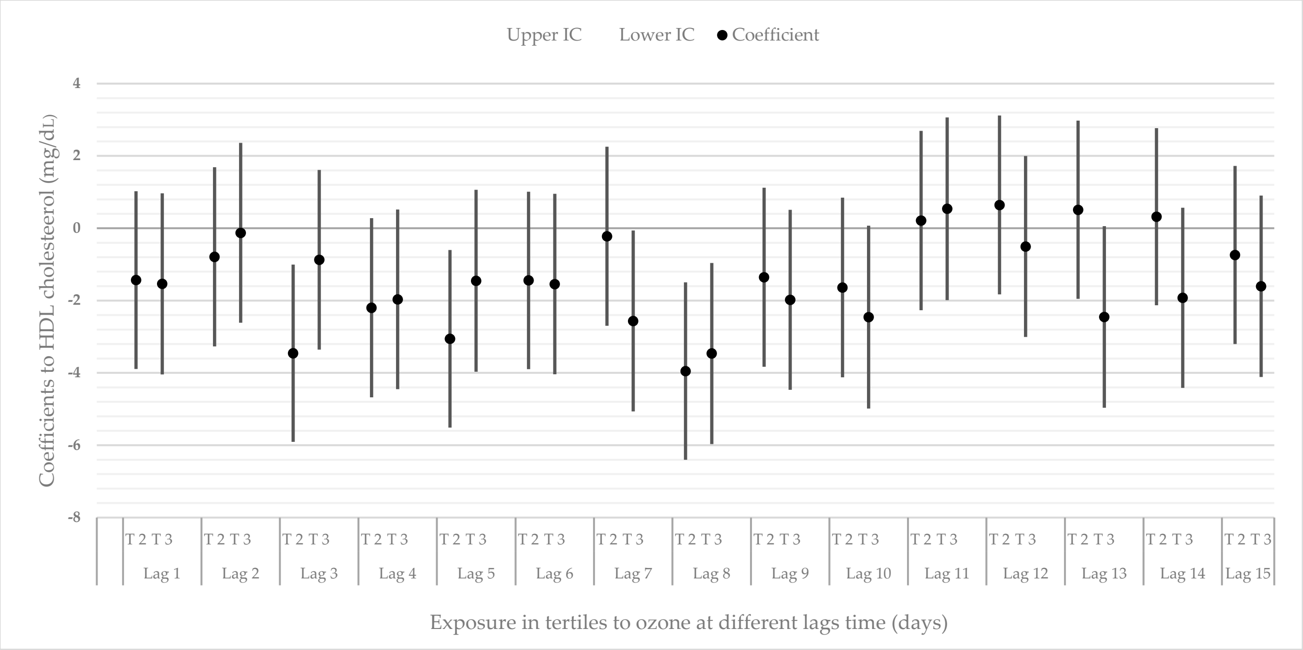

Figure S3. Association between HDL cholesterol (mg/dl) and short term exposure to ozono at differents lags of time (1 to 15 days) in adolescents obese, México City.

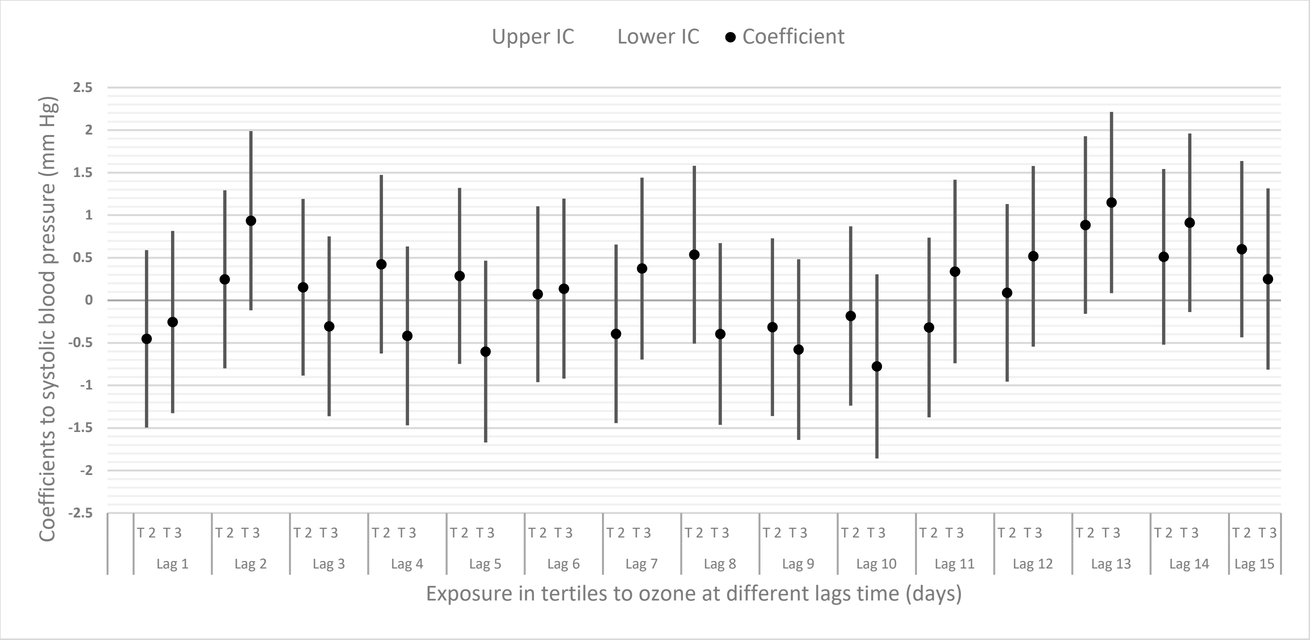

Figure S4. Association between systolic blood pressure (mmHg) and short term exposure to ozono at differents lags of time (1 to 15 days) in adolescents obese, México City.

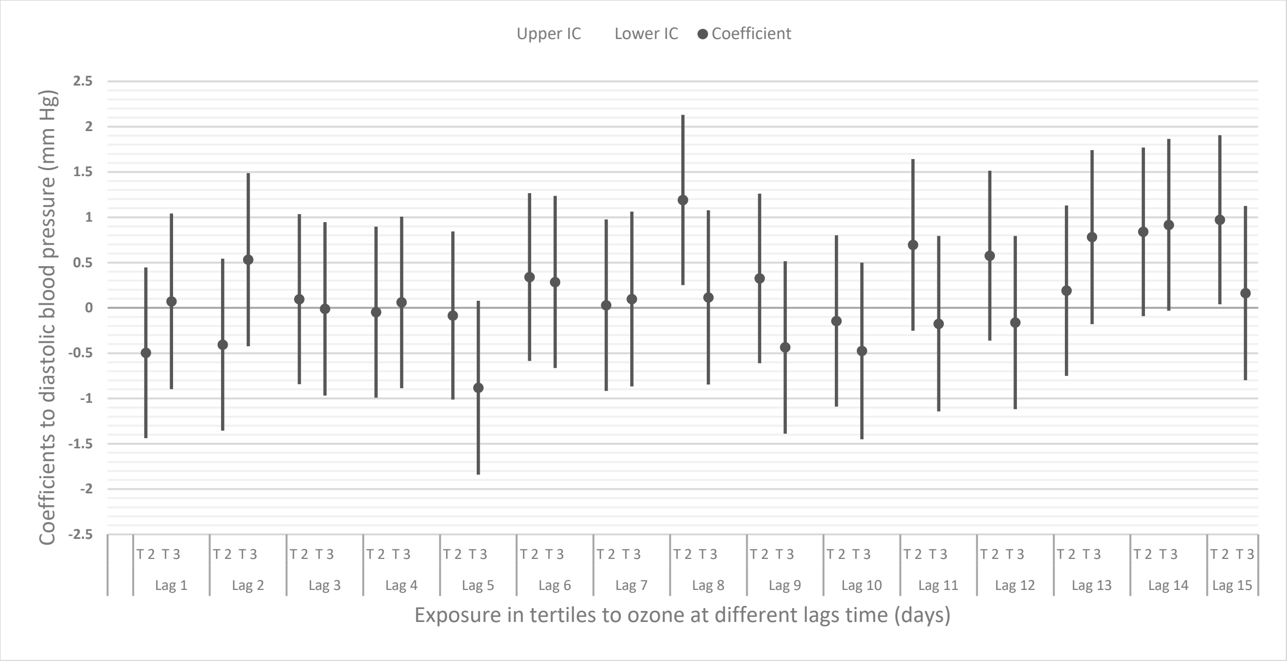

Figure S5. Association between diastolic blood pressure (mm Hg) and short term exposure to ozono at differents lags of time (1 to 15 days) in adolescents obese, México City.

Note : All models were carried using mixed effects model with random intercept adjusted by physical activity, BMI, antioxidant intake (Vitamin C and Vitamin E) and asthma presence** Ozone unit measurement was made by parts per million (ppm) and classified in tertiles (***Blood Pressure (B.P.). n=372 (Averages of evaluations per participant= 3. 4, 2 visits minimum and 16 visits minimum, with differences between each visit of 3 months minimum).
